# Artificial Intelligence and Blockchain Integration in Business: Trends from a Bibliometric-Content Analysis

**DOI:** 10.1007/s10796-022-10279-0

**Published:** 2022-04-12

**Authors:** Satish Kumar, Weng Marc Lim, Uthayasankar Sivarajah, Jaspreet Kaur

**Affiliations:** 1 Department of Management Studies, Malaviya National Institute of Technology, Jaipur, Rajasthan 302017 India; 2 Faculty of Business, Design and Arts, Swinburne University of Technology, Jalan Simpang Tiga, 93350 Kuching, Sarawak Malaysia; 3 School of Business, Law and Entrepreneurship, Swinburne University of Technology, John Street, Hawthorn, Victoria 3122 Australia; 4 School of Management, Faculty of Management, Law and Social Sciences, University of Bradford, Richmond Road, Bradford, BD7 1DP UK

**Keywords:** Artificial intelligence, Blockchain, Business, Fourth industrial revolution, IR 4.0, Integration, Trends

## Abstract

Artificial intelligence (AI) and blockchain are the two disruptive technologies emerging from the Fourth Industrial Revolution (IR4.0) that have introduced radical shifts in the industry. The amalgamation of AI and blockchain holds tremendous potential to create new business models enabled through digitalization. Although research on the application and convergence of AI and blockchain exists, our understanding of the utility of its integration for business remains fragmented. To address this gap, this study aims to characterize the applications and benefits of integrated AI and blockchain platforms across different verticals of business. Using bibliometric analysis, this study reveals the most influential articles on the subject based on their publications, citations, and importance in the intellectual network. Using content analysis, this study sheds light on the subject’s intellectual structure, which is underpinned by four major thematic clusters focusing on supply chains, healthcare, secure transactions, and finance and accounting. The study concludes with 10 application areas in business that can benefit from these technologies.

## Introduction

Artificial intelligence (AI) is a technology that can perform complex tasks that require human intelligence, and it holds the potential of exceeding human capabilities (Agarwal et al., 2020; Pandl et al., 2020). AI is one of the main drivers of industrial development as it promotes the integration of emerging technologies in the Fourth Industrial Revolution (IR 4.0) (Goodell et al., 2021; Lim, 2019; Zhang et al., 2021), such as blockchain (Ehrenberg & King, 2020), cryptocurrency (Li & Whinston, 2020), cloud computing (Hsu, 2020), and internet of things (IoT) (Ghaleb et al., 2021). Indeed, the massive amount of data generated by IoT devices, social media, and web applications has fueled the proliferation of AI, wherein the data is utilized in the training of machine learning algorithms (Dinh & Thai, 2018). However, some concerns associated with AI exists. Specifically, privacy has become a critical concern as a result of a series of leaks and misuse of personal data. The Facebook scandal in which millions of users were targeted without consent by Cambridge Analytica, a third-party political firm, is one such example. Other growing concerns with AI include explainability and trustworthiness as the technology does not interact or speak with human users and thus cannot be verified or trusted (Dinh & Thai, 2018).

Likewise, blockchain has gained increasing attention as a technology with a wide range of applications in various fields (Dinh & Thai, 2018; Liu et al., 2019a). Blockchain, which became popular after the emergence of bitcoin in 2008 (Nakamoto, 2008), has remained as a disruptive technology that transforms the way we interact, trace transactions, and automate payments, among others (Roszkowska, 2020). Indeed, blockchain has opened the door of opportunities that enables the direct transference of value between its users in a secure and trusted manner. Moreover, with the execution of smart contracts, the checking of approvals and compliances can become simpler, as blockchain is a shared database that is synchronized across multiple sites, and thus, such activities can be agreed on by each participant in a distributed network (Soleymani & Paquet, 2020). The data in blockchain is stored in blocks with hash values and timestamps, wherein the blocks are created based on a consensus protocol such as a *proof of work* or *a proof of stake* (Dinh & Thai, 2018)—proof of stake is less expensive than proof of work due to higher energy efficiency (Karafiloski & Mishev, 2017). More importantly, all transactions are cryptographically signed on blockchain, wherein all mining nodes that hold a copy of the entire ledger verify every single transaction on the blockchain (Nakamoto, 2008; Zheng et al., 2017). Therefore, blockchain is cost effective and secure as it eliminates the need for a centralized authority to verify the transactions (Karafiloski & Mishev, 2017; Zhu et al., 2021).

The developments of AI and blockchain has propelled their integration to revolutionize the next digital generation ignited by IR 4.0. According to Salah et al. (2019), blockchain can offer explainability, privacy, and trust to AI-based applications, whereas AI can enhance scalability and security while resolving the personalization and governance issues for blockchain-based technologies. As indicated in Table 1, AI and blockchain are technically different in various ways, but they can be used to overcome the shortcomings of each other. In that sense, AI and blockchain are the *yin* and *yang* of digital business, wherein AI helps the business to understand, recognize, and decide, whereas blockchain supports the business to execute, verify, and record (Morrison, 2016).

**Table 1:** Artificial intelligence and blockchain characteristics

Artificial intelligence (AI)	Blockchain
AI is driven by centralized infrastructure.	Blockchain is predicated on decentralized and distributed infrastructure.
AI decisions are made by machine learning systems that are unexplainable to human users, and thus, lacks transparency.	Blockchain can be explained to human users and is transparent as it can be tracked.
AI is probabilistic.	Blockchain is deterministic.
AI models and adapt over time.	Blockchain is immutable.

In practice, the convergence of AI and blockchain has brought many new opportunities (Makarius et al., 2020). In healthcare, blockchain enables the secure storage of patient data. When access is granted, health professionals gain insights from this data through the patterns churned by AI. Noteworthily, their joint application has helped the healthcare industry to navigate the COVID-19 crisis (Fusco et al., 2020). BurstIQ, a blockchain-based company providing data solutions for the healthcare industry, is an innovative example that provides a health wallet based on blockchain, AI, and big data to manage patient data. The wallet provides patient health records to health professionals so that they can learn more about the patient’s health condition as and when necessary (Daley, 2019). The convergence of the two technologies is also re-inventing the financial services industry by increasing the speed of transactions and enabling trust among transacting parties (Soleymani & Paquet, 2020). Similarly, AI and blockchain has transformed supply chains by digitizing traditional paper-based processes, enabling trustworthy data sharing, and facilitating automated transactions (Yong et al., 2020). IBM’s food trust blockchain technology and AI platform is an example that has assisted small scale coffee and cocoa farmers to speed up their transactions and improve their farming decisions (Barbano, 2017).

In literature, the integration of AI and blockchain has been reported to produce wide-ranging applications for different sectors such as autonomous vehicles, finance, smart cities, and 6G networks. Dinh and Thai (2018) offered a conceptual articulation of integrating the two technologies and organizes their benefits through two categories in the form of *AI for blockchain* and *blockchain for AI*, whereas Salah et al. (2019) presented insights on blockchain applications for AI through a critical review of the extant literature, and thus, covers only the latter but not the former category. More recently, Pandl et al. (2020) sought to address this knowledge gap as they shed light on the convergence of AI and distributed ledger technology (or blockchain) using a systematic literature review, and thus, covering both categories. However, none of these reviews unpacked the applications of integrating AI and blockchain for business, which is the key activity underpinning economic activity and growth of a country.

Today, the language of business involves compound concepts such as dematerialization, disintermediation, and designing and producing goods on demand (Kumar, 2019). In this regard, success in the next industrial era requires companies to reconfigure their business models in ways where technology becomes central to their operations in order to address these changing demands in the future of work and marketplaces. AI and blockchain are powerful technologies that are well positioned for this endeavor as they hold the potential to reform existing processes for greater efficiency and seamlessness. Indeed, the paradigm of organizations today is transitioning from a hierarchical to a self-organizing model (Subic et al., 2020). While AI and blockchain have initially focused on the finance sector, companies today have come to realize its potential for other sectors, including agriculture, healthcare, logistics, manufacturing, and supply chains. (Pandl et al., 2020). Yet, no study, to date, have shed light on the peculiarities and opportunities for AI and blockchain integration specifically for business through a scientific consolidation of knowledge, which is arguably important for both future research (e.g., what else should we know) and practice (e.g., what should we do) in the field.

This article aims to address the identified research gap by exploring the utility of AI and blockchain integration for business. In particular, this article endeavors to identify the research peculiarities of the field and to explain how the integration of AI and blockchain can benefit different verticals of business in the era of IR 4.0. To do so, this article carries out a study using a bibliometric-content analysis to identify the publication productivity and the intellectual structure of the field. Following the convention of past bibliometric studies (Donthu et al., 2021b, c; Kumar et al., 2021a, b, c; Lim et al., 2022b, c) and the aims of the article, this study addresses the following research questions (RQs):

- **RQ1.** What is the *publication productivity* of research on AI and blockchain integration for business? The answer to this research question offers insights on the *number* and *growth* of scientific articles in the field.
- **RQ2.** What are the *most influential articles* on AI and blockchain integration for business? The answer to this research question enables academic scholars and business professionals to locate the *key* and *seminal* articles in the field.
- **RQ3.** What are the *most prominent topics and themes* on AI and blockchain integration for business? The answer to this research question provides a comprehensive understanding on the *content* in the field’s body of knowledge.
- **RQ4.** What are the *most promising areas* for business to apply AI and blockchain integration? The answer to this research question guides business professionals on the *application* of AI and blockchain integration.

This article makes several noteworthy contributions. First, this article sheds light on AI and blockchain integration specifically for business, which to date, remains to no avail, wherein such insights are arguably important given that business is the engine of the economy, and thus, their transformation to remain relevant in the digital era is a high priority. Second, this article elucidates the applications and benefits that these technologies can offer to business, which represent important takeaways for both academia and industry. Third, this article represents the first study to perform a bibliometric analysis on AI and blockchain integration, which is an important endeavor to help interested scholars and professionals to gain a scientific overview of existing research in the field (Donthu et al., 2021a). While prior literature reviews on AI and blockchain avail, they remain limited in several ways. For example, the literature reviews by Omohundro (2014), Karafiloski and Mishev (2017), Dinh and Thai (2018), and Salah et al. (2019) are critical rather than systematic in nature, and thus, they are not replicable due to the absence of a review protocol and remain limited to the selective debates that they have deliberately chose to focus. The literature review by Pandl et al. (2020), though systematic, remains limited to manual, subjective insights. The use of bibliometric analysis herein this review can overcome the shortcomings of prior reviews as this review technique is predicated on statistical analysis on objective data (e.g., authorship, publication, citation) (Donthu et al., 2021a), and thus, represents a noteworthy step forward and a seminal contribution to the field. Table 2 presents the research gaps addressed by this study and maps the contribution of research questions toward the research gaps. The comparison of the focus, method, and contribution of the present review against prior reviews, which further accentuates the novelty of the present review, is presented in Table 3.

**Table 2:** Mapping of research gaps, research questions, and research contributions

Research gap	Research question	Research contribution
The paucity of objective retrospections on the extant literature of AI-blockchain integration and its business application.	RQ1. What is the publication productivity of research on AI and blockchain integration for business?	• Insights on year-wise distribution of publications. • Most of publications appeared in 2019 and 2020, which indicates an emerging field of research with ample scope for further investigation.
	RQ2. What are the most influential articles on AI and blockchain integration for business?	• Insights on highly cited articles. • Enable academic scholars and business professionals interested in AI-blockchain integration for business to easily key and seminal readings in the field.
The absence of a review that objectively investigates the emerging topics and themes in research on the integration of AI and blockchain for business application.	RQ3. What are the most prominent topics and themes on AI and blockchain integration for business?	• The top 20 topics in the field are revealed through a keyword co-occurrence analysis. • The four major themes of research in the field are revealed through bibliographic coupling.
The dearth of studies investigating the different areas in business that benefitted from the application of integrated AI-blockchain platforms.	RQ4. What are the most promising areas for business to apply AI and blockchain integration?	• 10 application areas for AI-blockchain integration in business are identified. • The value-added contribution of AI-blockchain integration to the processes, products, and/or services in each of the 10 application areas are provided.

**Table 3:** Comparison of the present study against existing literature reviews in the field

Study	Focus	Method	Contribution
Omohundro (2014)	Application of AI in smart contracts and cryptocurrencies.	Critical review	• How smart contract and cryptocurrencies can provide infrastructure to ensure that AI systems follow stipulated safety and legal regulations.
Karafiloski and Mishev (2017)	Resolving big data challenges through blockchain solutions.	Critical review	• How blockchain can be used for organizing, storing, and processing big data. • The role of blockchain for user authentication, recording data access history, and restricting user access based on need.
Dinh and Thai (2018)	Conceptual ideas of AI and blockchain integration.	Critical review	Two main perspectives: • How AI can be used for blockchain. • How blockchain can be used for AI.
Salah et al. (2019)	Research challenges on the use of blockchain for AI.	Critical review	Using blockchain for AI improves: • Data security. • Business process efficiency. • Trust on robotic decisions. • Collective decision making. • Decentralized intelligence.
Pandl et al. (2020)	Convergence of AI and distributed ledger technology (or blockchain).	Systematic literature review	Insights on the different ways in which: • AI can benefit distributed ledger technology. • Distributed ledger technology can benefit AI.
The present study	Applications of integrated AI and blockchain platforms in business.	Bibliometric-content review	Insights on AI and blockchain integration in business in terms of: • Publication productivity by year. • Most influential articles. • Prominent topics and co-occurrences. • Intellectual structure and its major thematic clusters. • 10 application areas.

The rest of the article is organized as follows. The article begins with a disclosure of the methodology guiding its study, followed by a detailed presentation of the results from a bibliometric-content analysis. The article concludes with key takeaways from the study, with limitations acknowledged and suggestions for future research offered at the end.

## Methodology

**Fig. 1:**
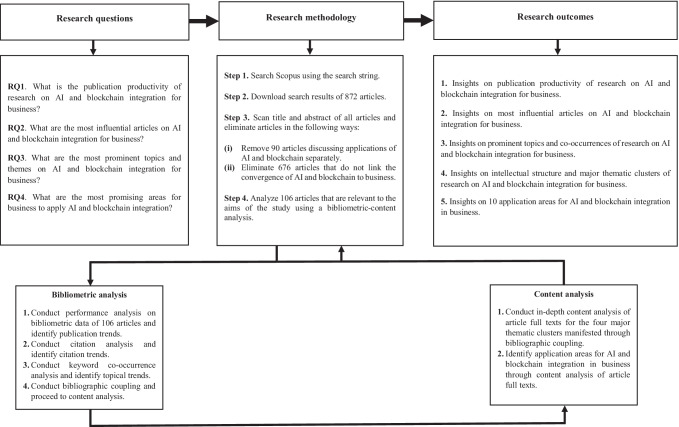
Research design and scheme of analysis

The present study adopts bibliometric analysis to determine the performance (e.g., publication productivity, most influential articles) and intellectual structure (e.g., topics, themes, application areas) of the literature on AI and blockchain integration in business. In essence, bibliometric analysis is a well-established scientific method for analyzing a body of literature, wherein bibliometric data (e.g., publication and citation information) are analyzed using quantitative tools (Pritchard, 1969; Donthu et al., 2021a; Mukherjee et al., 2021). The methodology is a recognized method of scientific enquiry having applications in various disciplines, including business (Donthu et al., 2021b, c; Kumar et al., 2021a, b, c; Lim et al., 2022b, c; Zupic & Čater, 2015). Noteworthily, many methods to review the literature avail (Lim et al., 2022a; Paul et al., 2021), but the bibliometric method is the most objective due to its reliance on a review protocol and quantitative analytical techniques—the other review methods either lack a review protocol (e.g., critical) and/or remain limited to subjective interpretations due to the absence of objective analysis techniques (e.g., thematic) (Donthu et al., 2021a; Lim et al., 2022a). Specifically, the present study adopts the four-step procedure for bibliometric analysis recommended by Donthu et al. (2021a), which includes *defining the aims and scope for study*, *choosing the techniques for analysis*, *collecting the data for analysis*, and *conducting the analysis and reporting the findings*. The overview of the study’s methodology is presented in Fig. 1.

### Defining the Aims and Scope for Study

The present study aims to shed light on the bibliometric and intellectual structure of the extant literature pertaining to AI and blockchain integration for business. The bibliometric structure encapsulates the publication productivity, whereas the intellectual structure pertains to the major topics and themes of research in the area under study. The scope for study is relatively large, as AI and blockchain are rich areas of research.

### Choosing the Techniques for Analysis

The present study employs a combination of bibliometric and content analysis techniques to map the development of the literature pertaining to AI and blockchain integration for business. In particular, the study conducts a performance analysis using a variety of bibliometric measures such as citations, cite score, impact factor, publication count, and *h*-index, as well as keyword co-occurrence analysis and bibliographic coupling to unpack the major topics and themes in the research field, which subsequently informs the authors’ reading of article full text for the content analysis.

### Collecting the Data for Analysis

To identify the articles and associated bibliometric and bibliographic data for analysis, the authors engaged in brainstorming with subject-matter experts on AI and blockchain to develop the keywords to be included for the search. In essence, the concept of “AI-enabled blockchain” is an amalgamation of two technologies, namely AI and blockchain. In order to ensure that both of these concepts are comprehensively captured by the search keywords, the authors included a search string for data search comprising of keywords covering the two technologies, as presented in Table 4.

**Table 4:** Search string and search results

Search string	Search results
(“Machine Learning”) OR (“AI”) OR (“Neural Network”) OR (“Artificial Intelligence”) OR (“Deep Learning”)	872 articles
AND	
(“Blockchain”) OR (“Block-chain”) OR (“Block chain”) OR (“Bitcoin”) OR (“Ethereum”) OR (“Hyperledger”) OR (“Cryptocurrency”) OR (“Smart contract”) OR (“Distributed Ledger Technology”) OR (“DLT”) OR (“Distributed Ledger”)	

The first part of the search string consists of terms related to AI (e.g., “artificial intelligence” and “machine learning”) and its most common applications (e.g., “neural network” and “deep leaning”). The second part of the search string comprises of keywords related to blockchain (e.g., “blockchain”, “distributed ledger”, and “hyperledger”) and its most common applications (e.g., “bitcoin”, “ethereum”, and “smart contract”).

The search is limited to document type (i.e., article and review), source type (i.e., journal), language (i.e., English), and year of publication (i.e., up to 2020). The search was conducted in February 2021. The data for the study is fetched from Scopus as it is the largest scientific database for peer-reviewed research publications (Bartol et al., 2014; Donthu et al., 2021a; Paul et al., 2021). The search of both search strings resulted in 872 articles.

In order to select the articles that are relevant for the study, the authors follow a two-step approach. In the first step after the results were returned from the Scopus search, two co-authors independently coded the articles (i.e., AI, blockchain, and AI and blockchain) before coming together with another co-author to collectively review, identify, discuss, and agree on the removal of 90 articles that discussed about the applications of AI and blockchain separately as they do not cover the “integration” of these technologies, which is the focus of the present study. In the second step, the same group of co-authors decided to eliminate 676 articles that did not link the convergence of AI and blockchain to business using the same process in the first step but with different codes (i.e., business, non-business). These articles explain only the technical aspects of the integration of AI and blockchain without inferring to how it brings cost-effectiveness, resilience, and flexibility to the business. After removing 676 articles, 106 articles that are relevant to the aims of the study were retained for analysis. The overall coding agreement for articles across the two steps was at 96% between two co-authors and a majority vote was implemented to resolve disagreements with the inclusion of another co-author in line with La Paz et al. (2020). Table 5 presents the inclusion and exclusion criteria used for shortlisting the articles relevant to the study.

**Table 5:** Inclusion and exclusion criteria and procedure

Steps	Articles excluded	Reason for exclusion	Articles included	Reason for inclusion
Step 1. Scopus search	0	Not a filtering step.	872	Results returned from search.
Step 2: Read title and abstract of articles	90	Articles do not cover the integration of AI and blockchain—only either one of the two technologies, not both.	782	Articles cover the integration of AI and blockchain.
Step 3: Read full text of articles	676	Articles focus only on core technical aspects of integrated AI and blockchain platforms, and do not explicitly explain the benefits of the technological integration to business functions.	106	Articles goes beyond core technical aspects, and explicitly discuss the applications and benefits of integrated AI and blockchain platforms in/for business.

### Conducting the Analysis and Reporting the Findings

The final step involves conducting the bibliometric-content analysis and reporting the findings from the analysis. To do so, the authors use VOSviewer (Van Eck & Waltman, 2010) and Gephi software to perform bibliometric analysis and to visualize its output in a network. Additionally, the authors use a Python code to create word clouds representing the main themes of bibliographic clusters to enrich the presentation of the findings, wherein Python 3.7 in combination with Jupyter Notebook were used to run the code and to generate the word clouds. The full text of each article in each major thematic cluster revealed through the bibliometric analysis was read and analyzed accordingly. The findings of the bibliometric-content analysis are reported in the next sections.

## Findings

Findings from the bibliometric-content analysis are presented based on the research questions that they address. In particular, findings pertaining to publication productivity, influential articles, prominent themes, and promising application areas are related to the first, second, third, and fourth research questions, respectively, and the sections are organized as follows.

### Publication Productivity

To answer RQ1—i.e., what is the publication productivity of research on AI and blockchain integration for business—the study analyzes the total publications in the field by year. The bibliometric data employed for the analysis is collected from Scopus. Bibliometric data is a form of big data consisting of information associated to scientific publications, such as publication (e.g., title, abstract, keywords, year) and citation (e.g., author, document, and journal citation count) information (Broadus, 1987; Donthu et al., 2021a).

**Fig. 2:**
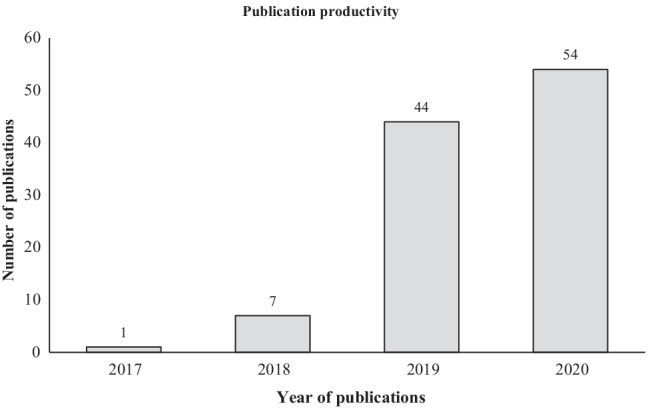
Annual distribution of publications on AI and blockchain integration for business

The number of publications on AI and blockchain integration for business is shown in Fig. 2. In particular, all publications in this domain are published between 2017 and 2020, with most publications appearing in 2019 and 2020, which reflects its status as an emerging field of research that has started to get popular recently. Indeed, AI and blockchain are two hallmark technologies of IR 4.0 that were introduced only in 2016, and thus, it is unsurprising that research of its integration have entered academia only in 2017, as initial research investment have focused on each technology independently, and proliferated since 2019, which signals that the integration of AI and blockchain are indeed important for business.

### Most Influential Articles

**Fig. 3:**
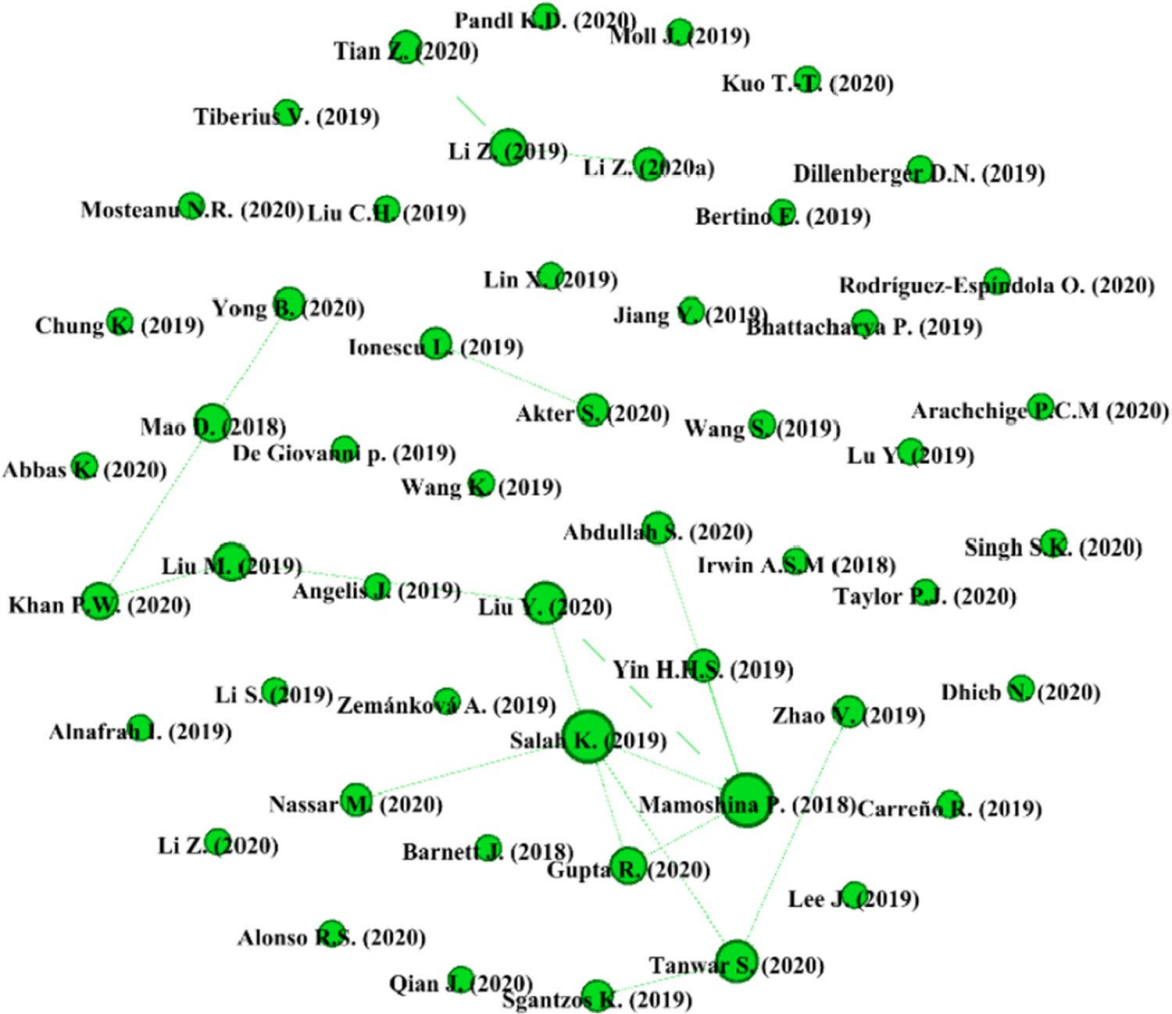
Citation network for publications on AI and blockchain integration for business

To answer RQ2—i.e., what are the most influential articles on AI and blockchain integration for business—the study conducts a performance analysis by means of citation network for the 106 articles under review. There are several metrics to measure the influence of a publication, but the most prevalent metric is citations (Ding & Cronin, 2011), wherein the influence of a publication is assessed through the number of times it is cited by other publications (Donthu et al., 2021a). VOSviewer and Gephi software were employed to derive the citation network between publications. Figure 3 presents the citation network among publications and Table 6 shows the leading original articles by the number of citations. Mamoshina et al.’s (2018) article on the convergence of AI and blockchain in healthcare tops the list with 102 citations, followed by Liu et al.’s (2019b) article on performance optimization for blockchain-enabled IoT systems and Mao et al.’s (2018) article on credit evaluation system based on LSTM and blockchain with 47 and 44 citations, respectively.

**Table 6:** Top cited original articles on AI and blockchain integration for business

Rank	Year	Title	Author(s)	Journal	TC
1	2018	Converging blockchain and next-generation artificial intelligence technologies to decentralize and accelerate biomedical research and healthcare	Mamoshina et al.	*Oncotarget*	102
2	2019	Performance optimization for blockchain-enabled industrial internet of things (IIOT) systems: A deep reinforcement learning approach	Liu et al.	*IEEE Transactions on Industrial Informatics*	47
3	2018	Credit evaluation system based on blockchain for multiple stakeholders in the food supply chain	Mao et al.	*International Journal of Environmental Research and Public Health*	44
4	2020	Machine learning adoption in blockchain-based smart applications: The challenges, and a way forward	Tanwar et al.	*IEEE Access*	39
5	2019	Blockchain adoption: A value driver perspective	Angelis and da Silva	*Business Horizons*	37
6	2020	BlockIoTIntelligence: A blockchain-enabled intelligent IoT architecture with artificial intelligence	Singh et al.	*Future Generation Computer Systems*	27
7	2020	Smart contract privacy protection using AI in cyber-physical systems: Tools, techniques and challenges	Gupta et al.	*IEEE Access*	27
8	2019	Regulating Cryptocurrencies: A supervised machine learning approach to de-anonymizing the bitcoin blockchain	Sun Yin et al.	*Journal of Management Information Systems*	26
9	2019	Machine learning based privacy-preserving fair data trading in big data market	Zhao et al.	*Information Sciences*	25
10	2019	A blockchain and automl approach for open and automated customer service	Li et al.	*IEEE Transactions on Industrial Informatics*	19
11	2019	An intelligent blockchain-based system for safe vaccine supply and supervision	Yong et al.	*International Journal of Information Management*	14
12	2020	Food traceability system from governmental, corporate, and consumer perspectives in the European Union and China: A comparative review	Qian et al.	*Trends in Food Science and Technology*	10
13	2020	Blockchain and machine learning for communications and networking systems	Liu et al.	*IEEE Communications Surveys and Tutorials*	9
14	2020	IoT-blockchain enabled optimized provenance system for food industry 4.0 using advanced deep learning	Khan et al.	*Sensors*	8
15	2020	Blockchain for explainable and trustworthy artificial inteavblligence	Nassar et al.	*Wiley Interdisciplinary Reviews: Data Mining and Knowledge Discovery*	8
16	2020	Transforming business using digital innovations: The application of AI, blockchain, cloud and data analytics	Akter et al.	*Annals of Operations Research*	avb
17	2020	Artificial intelligence implementations on the blockchain. Use cases and future applications	Sgantzos and Grigg	*Future Internet*	8
18	2019	Big data, blockchain, and artificial intelligence in cloud-based accounting information systems	Ionescu	*Analysis and Metaphysics*	7
19	2020	CrowdSFL: A secure crowd computing framework based on blockchain and federated learning	Li at al.	*Electronics*	5
20	2019	A blockchain-based evaluation approach for customer delivery satisfaction in sustainable urban logistics	Tian et al.	*International Journal of Production Research*	4

*TC*  total citations

Mamoshina et al. (2018) provides an overview of the innovative solutions in the field of biomedical research combining blockchain technology and AI. The authors describe the creation of distributed ledger of personal patient records, where patients can own and control their data. The authors also argue that the integration of blockchain and deep learning technologies creates a transparent and secure distributed personal data marketplace that can resolve the challenges experienced by regulators. Liu et al. (2019b) highlight the issues of data security and efficiency in industrial IoT (IIoT) applications that rely on centralized servers and suggest that blockchain combined with deep reinforcement learning (DRL) algorithm can optimize the performance of IIoT applications. The integration of blockchain and DRL helps to bring decentralization, security, and scalability to blockchain-enabled IIoT systems. Mao et al. (2018) introduce a credit evaluation system based on blockchain that collects credit evaluation data from traders through smart contracts, analyze it using a deep learning network called long short term memory, and provide evidence showing that a blockchain-powered credit evaluation system strengthens the efficiency of management and supervision in food supply chain. These publications are also among the most influential in the field, as seen through the links between their representing nodes with the nodes representing other publications in the field.

### Most Prominent Topics

**Fig. 4:**
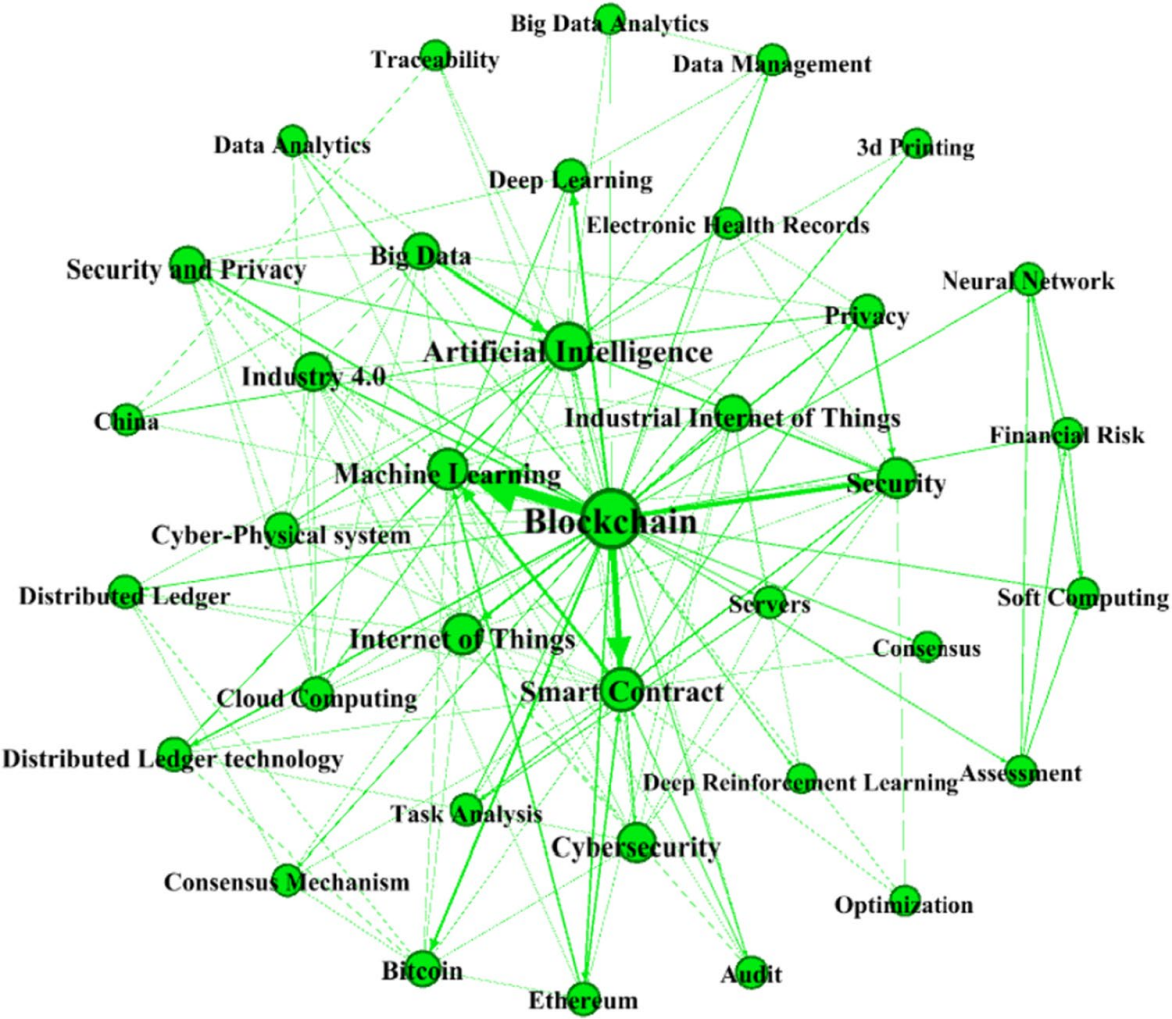
Topic network for publications on AI and blockchain integration for business

To answer the first part of RQ3—i.e., what are the most prominent *topics* and themes on AI and blockchain integration for business—the study performs a keyword co-occurrence analysis of author keywords listed in the 106 articles in the review corpus. Comerio and Strozzi (2018) suggest that the keywords that authors list in a publication reflect the topical coverage of that publication and their co-occurrence reflects the topical trends prevalent in the research field. The keyword occurrences, co-occurrences, and network are presented in Tables 7 and 8 and Fig. 4, respectively.

**Table 7:** Top keywords by frequency of occurrence

Keyword(s)	Occurrence(s)
Blockchain	74
Artificial intelligence	31
Smart contract	22
Machine learning	19
Internet of things	13
Security	7
Big data	6
Cybersecurity	6
Industry 4.0	5
Distributed ledger technology	5
Bitcoin	4
Deep learning	4
Ethereum	4
Privacy	4
Security and privacy	4
Industrial internet of things	4
Distributed ledger	3
Cyber-physical system	2
Assessment	2
Cloud computing	2

**Table 8:** Top keyword pairs by degree of co-occurrence

Keyword 1	Keyword 2	Weight
Blockchain	Machine learning	16
Blockchain	Smart contract	9
Blockchain	Security	7
Big data	Artificial intelligence	5
Smart contract	Machine learning	5
Blockchain	Bitcoin	4
Blockchain	Deep learning	4
Blockchain	Internet of things	4
Blockchain	Distributed ledger technology	3
Blockchain	Ethereum	3
Blockchain	Industry 4.0	3
Blockchain	Privacy	3
Blockchain	Security and privacy	3
Ethereum	Smart contract	3
Ethereum	Machine learning	3
Privacy	Security	3
Security	Artificial intelligence	3
3D printing	Blockchain	2
Blockchain	Assessment	2
Blockchain	Audit	2

The top 20 keywords based on their occurrences are presented in Table 8. *Blockchain* is the most prominent keyword in the list with 74 occurrences, followed by *artificial intelligence* (31), *smart contract* (22), *machine learning* (19), and *internet of things* (13). All these keywords are a reflection of the scope of publications in the dataset. More importantly, the keywords are consistent with the line of enquiry of the present study as they reflect the core technologies that can be employed and integrated for the automation and optimization of business in IR 4.0. The top 20 keyword pairs based on their co-occurrences are presented in Table 9. The keyword pairs are ranked on the basis of their strength or weight of association. The three most strongly associated keyword pairs are “*blockchain and machine learning*”, “*blockchain and smart contract*”, and “*blockchain and security*”. The sturdiest association of *blockchain* and *machine learning* signals that the dataset herein is representative of investigations that focus on the integration of AI and blockchain, wherein machine learning is the main form of AI relevant for business. The network among keywords is presented in Fig. 4. The size of the node represents the occurrence of the keyword, whereas the link between nodes and the thickness of that link represent the co-occurrence between keywords and the degree of that co-occurrence. Here, the topic network illustrates that *artificial intelligence*, *blockchain*, *machine learning*, *internet of things*, and *smart contract* are the most prominent nodes in the network that occur and co-occur most frequently together as well with other topical keywords, and thus, highlighting their relative importance in the field.

**Table 9:** Overview of the thematic clusters

Thematic cluster	Application	Source
Cluster 1. IR 4.0 and supply chains	A technology called PriModChain based on ethereum blockchain, smart contracts, federated machine learning, and differential privacy that enforces privacy and ensure trustworthiness of IIoT data.	Arachchige et al. (2020)
	A three-layer blockchain enabled cyber physical system (BCPS) that addresses the challenges of current manufacturing processes related to security, transparency, privacy, trustworthiness, and efficiency, among others.	Lee et al. (2019)
	A production capability evaluation system based on machine learning, IoT, and blockchain technology that improves the production efficiency of manufacturing systems.	Li et al. (2020)
	Mitigating the issues related to vaccine expiration and vaccine record fraud through an intelligent system based on blockchain and machine learning algorithms.	Yong et al. (2020)
	A digital platform applying AI, blockchain, edge computing, and IoT to enable resource monitoring and traceability in blockchain.	Alonso et al. (2020)
Cluster 2. Smart healthcare	Preventing forgery and misrepresentation of medical data using neural networks and error backpropagation blockchain framework.	Kim and Huh (2020)
	Preserving health data using GuardHealth, a technology based on consortium blockchain, smart contract, and graph convolution network, which eventually guarantees security of the system.	Wang et al. (2020)
	Enabling patients to control their own medical records through AI-mediated health data exchange on blockchain.	Mamoshina et al. (2018)
	A predictive system based on the combination of AI and blockchain to control the risk of COVID-19.	Fusco et al. (2020)
	A predictive model for intelligent storage allocation decision for health data using a machine learning classifier and a blockchain-based repository.	Uddin et al. (2020)
Cluster 3. Secure transactions	De-anonymizing the bitcoin blockchain through a supervised machine learning approach to identify bitcoin users involved in cybercriminal activities.	Sun Yin et al. (2019)
	A technology based on machine learning architecture identifying suspicious behavior of bitcoin users.	Irwin and Turner (2018)
	A unique solution to mitigate the risk of identity theft in the case of online transactions based on machine learning, blockchain, IoT, and online signature verification.	Jain et al. (2019)
	Mitigating the imperfections of secured transaction legal systems based on the integration of AI, IoT, and smart contract.	de las Heras Ballell (2017)
Cluster 4. Finance and accounting	Automation of accounting decisions using AI and blockchain transforming the day-to-day work of accountants.	Moll and Yigitbasioglu (2019)
	Automated and secure financial transactions through integration of AI, blockchain, big data, and cloud computing with finance.	Zheng et al. (2019)
	Financial portfolio management and optimization through DeepBreath, an application of convolution neural network and blockchain.	Soleymani and Paquet (2020)
	Prevention of corporate frauds using smart contracts and advanced AI.	Roszkowska (2020); Mao et al. (2018)
	Mitigating credit risk by integrating blockchain technology and the long short term memory (LSTM) deep learning.	Mao et al. (2018)

### Most Prominent Themes

**Fig. 5:**
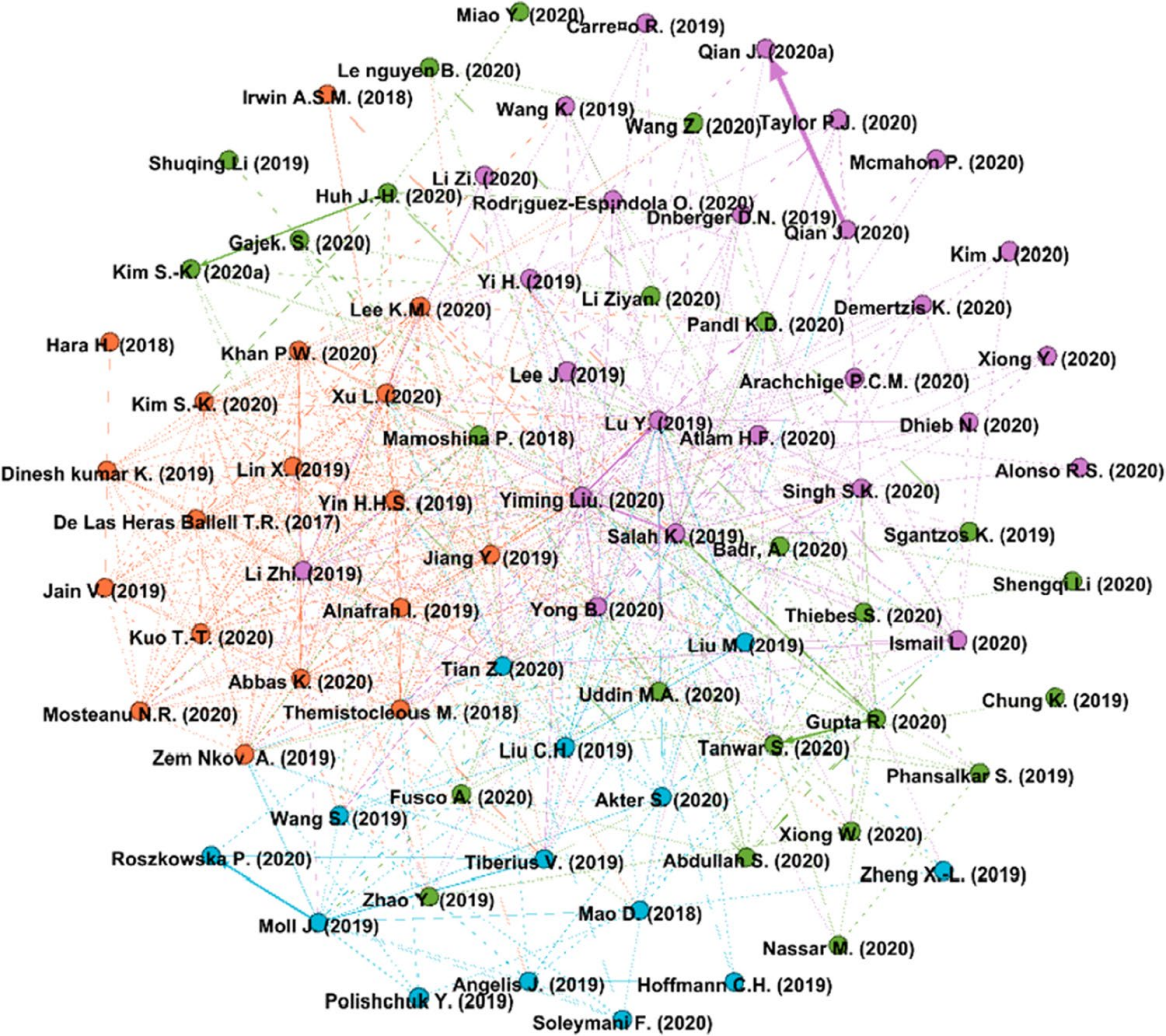
Bibliographic coupling map of thematic clusters. **Notes:** Purple = Cluster 1: IR 4.0 and supply chains. Green = Cluster 2: Smart healthcare. Orange = Cluster 3: Secure transactions. Blue = Cluster 4: Finance and accounting

To answer the second part of RQ3—i.e., what are the most prominent topics and *themes* on AI and blockchain integration for business—the study performs bibliographic coupling and content analysis of the 106 articles under review. Kessler (1963) coined the idea of bibliographic coupling and explained that scientific publications show intellectual associations through their referencing patterns, wherein scientific publications that cite similar sources form bibliographic couples to represent their intellectual associations. In that sense, bibliographic coupling is predicated on the assumption that publications that share common references are similar in their content (Kessler, 1963; Weinberg, 1974). The application of bibliographic coupling on the review corpus results in formation of four major thematic clusters that are adequately large enough to significantly represent more than 70% of publications on AI and blockchain integration for business in the corpus. The thematic clusters are presented in the bibliographic coupling map in Fig. 5, highlighting each cluster in a different color. Cluster 1 and cluster 2 are highlighted with purple and green nodes, whereas cluster 3 and cluster 4 are presented with orange and blue nodes, respectively. Specifically, two major thematic clusters are generic and thus transcend across industries (Clusters 1 and 3), whereas two major thematic clusters are industry specific (Clusters 2 and 4). Table 9 shows an overview of the technologies discussed in each thematic cluster.

The clusters are also analyzed using content analysis and the findings are presented based on their respective size, which is derived based on the associated number of publications.

#### Cluster 1: IR 4.0 and Supply Chains

**Fig. 6:**
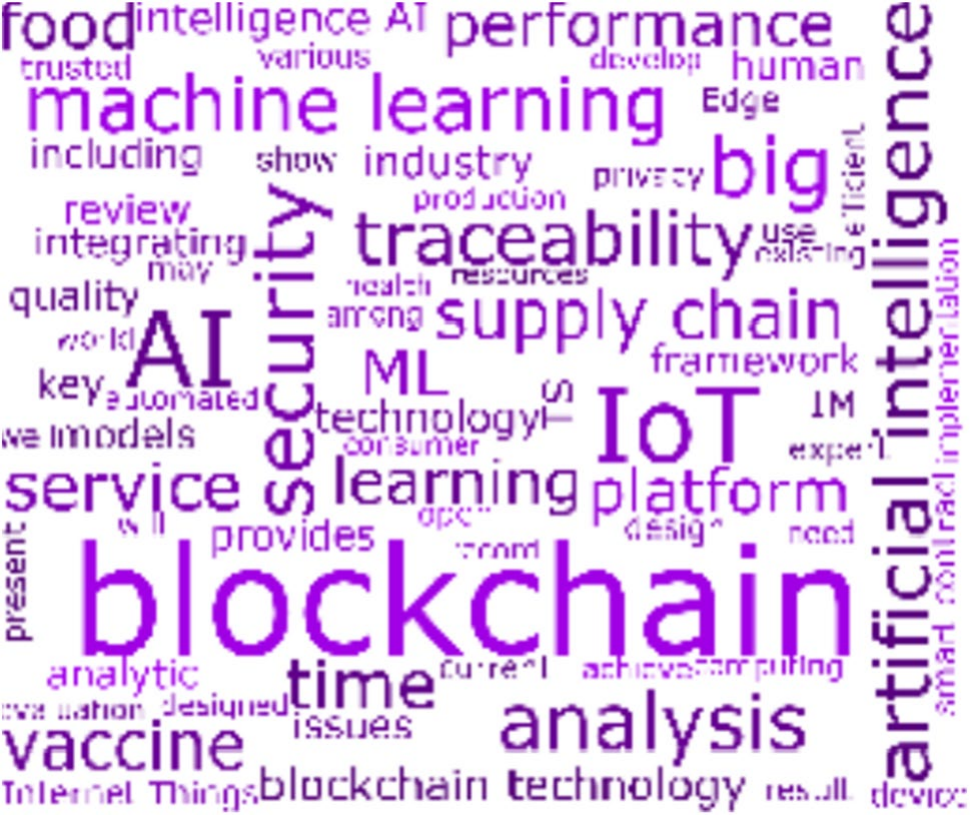
Cluster 1 word cloud on IR 4.0 and supply chains

Cluster 1 consists of 25 articles that have been cited 471 times. The articles in this cluster discuss about the developments of IR 4.0, wherein the concepts of *artificial intelligence*, *blockchain*, *supply chain*, and *traceability* appear prominently in the cluster’s word cloud in Fig. 6. Indeed, the topical concepts resonate to the integration of AI and blockchain as the prominent technologies that characterize the latest industrial revolution and that facilitate the automation business. Though IR 4.0 has created numerous opportunities to improve operations (Chen et al., 2022; Liu et al., 2019a; Rodríguez-Espíndola et al., 2020), the articles that organically manifested in this cluster through bibliographic coupling are focused on the optimization of supply chains, which is an activity that transcends across different industries.

Arachchige et al. (2020) describes IIoT as a driving force for IR 4.0 in business and explains how IIoT utilizes machine learning to manage large volumes of IIoT data that is prone to privacy adversarial attacks. Machine learning analyzes massive quantities of sensitive data produced by IIoT-based systems. There are high chances that machine learning models can compromise the confidential data to adversaries. The authors propose a *PriModChain* that amalgamates ethereum blockchain, smart contracts, federated machine learning, and differential privacy in order to enforce privacy and thus ensure trustworthiness of IIoT data. The privacy of the input data is strengthened using differential privacy whereas smart contracts offer transparency to the proposed technology (Ehrenberg & King, 2020; Han et al., 2021).

IR 4.0 has also led to enormous improvement in production efficiency in manufacturing systems. Cyber physical systems (CPS) combine various technologies such as AI, cloud computing, deep learning, and edge computing to control physical processes in manufacturing and to supervise the operations of self-organizing processes (Lu, 2017). However, CPS depends on third-party trust operations and centralized industrial networks due to which manufacturing processes suffer problems such as security, transparency, privacy, trustworthiness, and efficiency. Lee et al. (2019) proposes a three-layer blockchain enabled cyber physical system (BCPS) for to address these issues.

Additionally, a technically sound evaluation system can improve the production efficiency of manufacturing systems. Companies choose suppliers based on their production capability. However, conventional production capability evaluation systems are based on a centralized approach where there is limited sharing of performance data. Li et al. (2020) proposes a production capability evaluation system based on machine learning, IoT, and blockchain technology that can collect data in real time to support an automated enterprise production capability evaluation mechanism. In this instance, blockchain technology facilitates fair and automatic data trading through open and decentralized data storage.

Nonetheless, supply chains are vulnerable to different types of security and privacy threats—for example, in vaccine supply chains, issues such as vaccine record fraud and vaccine expiration are prevalent. Yong et al. (2020) highlight such issues and propose an intelligent system in which blockchain technology enables vaccine supervision, smart contracts detect expired vaccines, and machine learning algorithms provide vaccine recommendation information to different users. Similarly, in the dairy industry, it is important to provide detailed product information to consumers to ensure the quality and safety of the product. In order to enable traceability and resource monitoring in the value chain, Alonso et al. (2020) introduce a platform that applies AI, blockchain, edge computing, and IoT. The technology monitors the state of feed grains and dairy cattle in real time and ensure the traceability of production processes. Finally, Rodríguez-Espíndola et al. (2020) highlight the challenges in the flow of products, information, and financial resources in humanitarian supply chains and explain how the integration of AI, blockchain, and 3D printing can solve such problems.

#### Cluster 2: Smart Healthcare

**Fig. 7:**
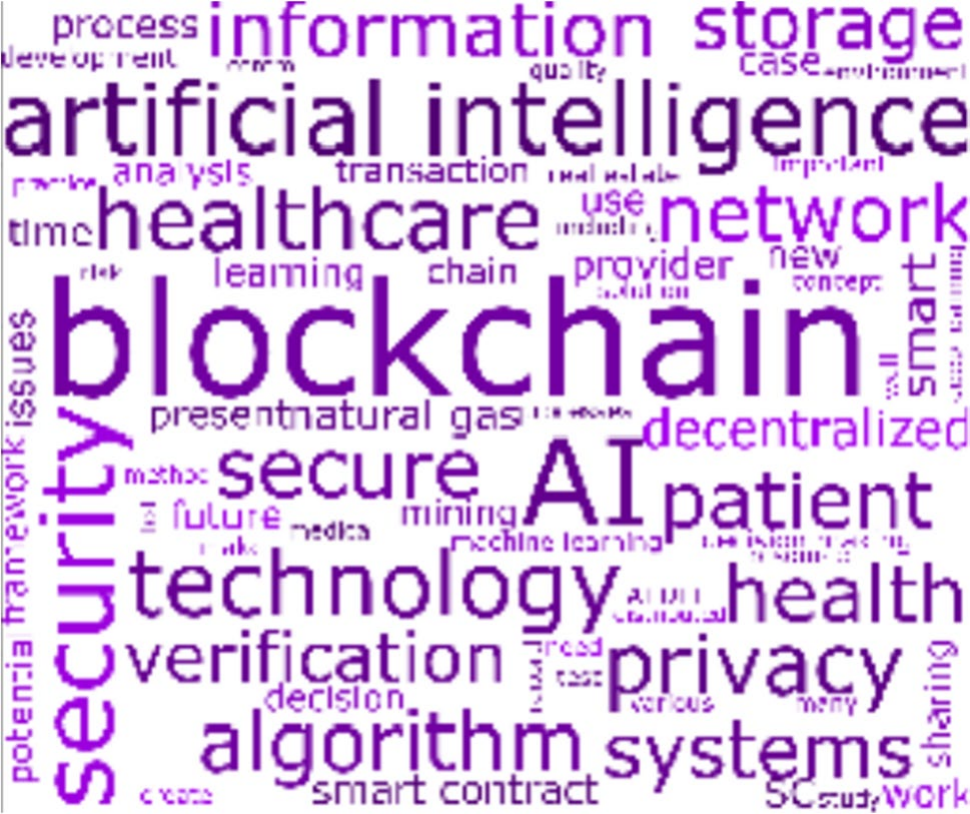
Cluster 2 word cloud on smart healthcare

Cluster 2 comprises 24 articles that have been cited 259 times. The main focus of the articles in this cluster is on the healthcare industry, wherein the concepts of *artificial intelligence*, *blockchain*, *health*, *healthcare*, and *patient* appear prominently in the cluster’s word cloud in Fig. 7.

In healthcare industry, the combination of AI and blockchain can help to address the issues related to medical data privacy (Klinker et al., 2019). Kim and Huh (2020) shed light on a medical information system using AI and blockchain technology. The paper shows the performance verification of the system using error backpropagation blockchain framework, wherein the proposed technology guarantees the integrity of the system by preventing forgery and misrepresentation of medical data using neural networks.

Wang et al. (2020) propose a scheme for preserving health data known as GuardHealth, which is based on consortium blockchain and two smart contracts for data storage and data sharing. The system provides data privacy and security by encrypting health data before uploading it to the cloud service provider by using proxy re-encryption. The proposed scheme also introduces a malicious node detection approach through graph convolution network that can build trust and guarantee security of the system.

Mamoshina et al. (2018) introduce the concept of AI-mediated health data exchange on blockchain and explain how blockchain technology and AI can enable patients to control their own medical records. The authors shed light on the application of transfer learning techniques and recurrent neural networks to the blockchain-enabled personal data marketplace that can further lead to various types of predictive analysis about the patient’s health, wherein the results of the predictive analysis can be helpful to pharmaceutical and insurance companies. The authors also propose a personal data-driven economy in which patients have complete control over their data and manage the access privileges to protect their data privacy. The system allows patients to be rewarded for generating new data and offering the data for research and commercial use.

More recently, Fusco et al. (2020) propose a predictive system based on the combination of AI and blockchain that can contribute to controlling the risk of COVID-19 in a national territory. Here, the predictive system can be autonomously and constantly updated with clinical data of patients, which in turn, can create big data suitable for informing health policy at the national level, and thus, demonstrating how business can support government in times of crisis.

Finally, with the exponential growth in digital health data and the increase in the variety of data repositories, Uddin et al. (2020) present a predictive model for intelligent storage allocation decision for health data, wherein a machine learning classifier maps the type of health data with the features of storage repositories before allocating that data to the blockchain-based repository. Similarly, Badré et al. (2020) introduce the concept of shared decision making in integrated health services and propose a decentralized patient assignment system that uses machine learning, blockchain technology, and integer programming to improve the coordination among healthcare providers and patients.

#### Cluster 3: Secure Transactions

**Fig. 8:**
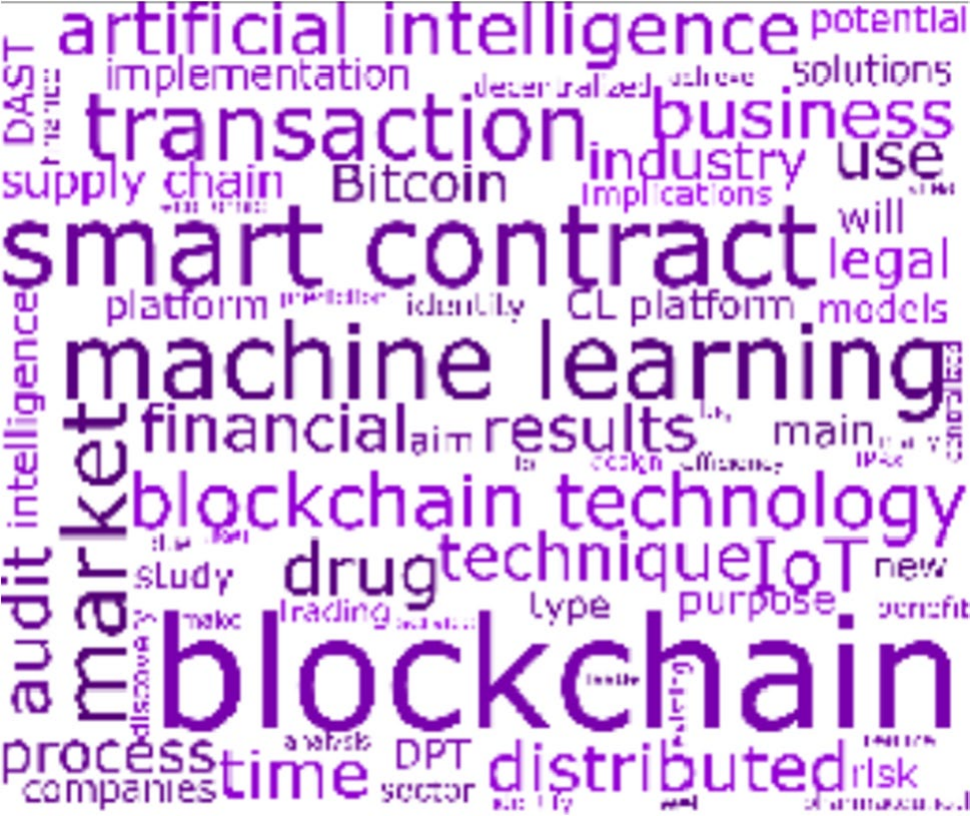
Cluster 3 word cloud on secure transactions

Cluster 3 contains 18 articles with 119 citations. The articles in this cluster encapsulate the risks associated with online transactions and the equivalent solutions to make such transactions secured, wherein the concepts of *blockchain*, *business*, *finance*, *machine learning*, and *transactions* appear prominently in the cluster’s word cloud in Fig. 8. In this cluster, it is observed that the high level of anonymity of cryptocurrencies makes them a fertile ground for illicit activities (Sun Yin et al., 2019). Indeed, law enforcement agencies have experience challenges in tracking down the identity of illegal cryptocurrency users and their transactions (Irwin & Turner, 2018).

To address the issue of intentional illicit transactions, Sun Yin et al. (2019) propose a method to de-anonymize bitcoin blockchain by employing a supervised machine learning approach, wherein the method helps to identify high risk bitcoin users and their potential cybercriminal activities. The author uses gradient boosting algorithm to uncover the anonymity of the parties.

Similarly, Irwin and Turner (2018) suggest an optimal solution based on a machine learning architecture that has predictive capabilities, wherein the solution enables predictive policing by collecting and analyzing data from bitcoin blockchain. The proposed technology collects data from bitcoin blockchain and identifies suspicious behaviors of bitcoin users by applying search criteria matching, indexing, and clustering on data. Moreover, the system encourages data sharing between cyber security organizations, the FinTech industry, and law enforcement and financial intelligence units, which will eventually enable law enforcement agencies to trace the identity of illegal bitcoin users.

To address the issue of unintentional illicit transactions, Jain et al. (2019) propose a unique solution to mitigate the risk of identity theft in the case of online transactions. Their system, which is founded on the amalgamation of machine learning, blockchain, IoT, and online signature verification, performs cryptocurrency transactions without using a private key. The verification of the transaction is done through dynamically generated handwritten signature, which is performed through specialized pen implanted with accelerometer and gyroscope. The handwritten signatures are authenticated through dynamic time warping algorithm.

Finally, de las Heras Ballell (2017) points out the imperfections of secured transaction legal systems and suggests that the integration of AI, IoT, and smart contract can remedy these imperfections and make the system more effective and secure. In secured transactions, the monitoring of collaterals bears significant transaction cost. Smart contracts and IoT can enable creditors to monitor encumbered assets in a cost-effective way, directly supervising compliance and instantly detecting infringement. Additionally, AI solutions embedded in smart contracts can mitigate the risk of funds recovery in case of insolvency, earmarking potential risk of obligations not being fulfilled due to unexpected events and making it technically impossible not to comply with agreed contractual terms. Therefore, the deployment of smart contract with the decision-making process of artificial technology and data collected by IoT solutions can enable a comprehensive contractual framework for authentic transactions.

#### Cluster 4: Finance and Accounting

**Fig. 9:**
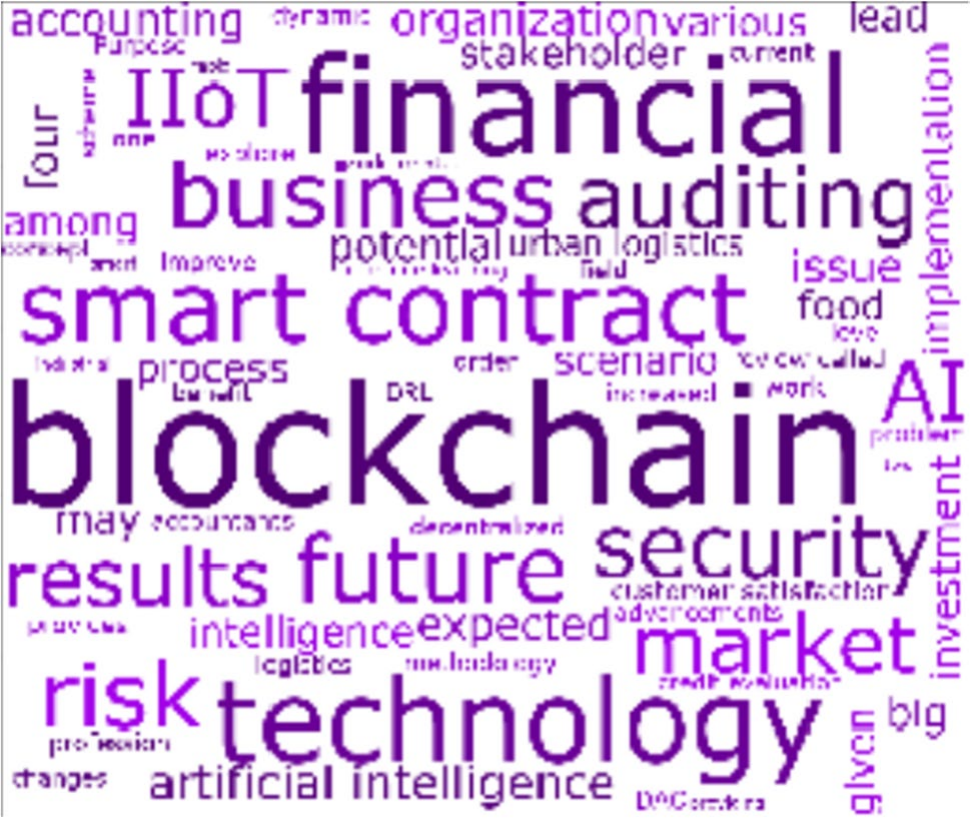
Cluster 4 word cloud on finance and accounting

Cluster 4 is the smallest cluster with 14 articles that have been cited 240 times. The articles in this cluster concentrate on the finance and accounting industry, wherein the concepts of *accounting*, *auditing*, *blockchain*, *business*, *financial*, and *smart contract* appear prominently in the cluster’s word cloud in Fig. 9.

In this cluster, it is observed that the integrated application of AI and blockchain can improve the processes in the industry. The use of information technologies can automate accounting decisions to a large extent and transform the everyday work of accountants (Moll & Yigitbasioglu, 2019). Zheng et al. (2019), who explain the three stages of technology-driven financial upgrading, shed light on FinTech 3.0, which involves the integration of technologies such as AI, big data, blockchain, and cloud computing with finance to achieve automated and secured financial transactions leading to improved service efficiency and reduced cost. Similarly, Soleymani and Paquet (2020) introduce an online framework for financial portfolio management and optimization using a deep reinforcement learning called DeepBreath. The convolutional neural network is used to implement investment policy based on which assets are reallocated in the portfolio to increase the return on investment, and the blockchain technology is used to reduce the settlement risk occurring due to delay between acquisition of asset and its payment.

In addition, the articles in this cluster explain how integrated AI and blockchain platforms can help to tackle problems such as corporate frauds and credit risk. Roszkowska (2020) discusses corporate frauds caused by the failure of the auditor to detect such frauds and argues how FinTech offers an effective solution to such issues, wherein the combined implementation of blockchain, smart contracts, and advanced AI solutions can overcome the deficiencies of financial reporting and auditing. Similarly, Mao et al. (2018) highlight the problem of credit risk caused by the information asymmetry between traders and propose a credit evaluation system that is implemented by integrating blockchain technology and the long short term memory (LSTM) deep learning network. This system uses smart contract to collect credit evaluation data and analyze it using LSTM. Indeed, the proposed system is an exemplar of how the integration of AI and blockchain can help to ensure the authenticity of information about credit evaluation and the verification of ensuing financial transactions by traders.

### Areas for AI and Blockchain Integration in Business

To answer RQ4—i.e., what are the most promising areas for business to apply AI and blockchain integration—the study scans the review corpus using a content analysis and the ensuing findings are presented in Table 10, wherein 10 promising areas for potential application of these hallmark IR 4.0 technologies are earmarked and explained with application exemplars and their associated technologies, value, and source.

**Table 10:** Promising areas for AI and blockchain integration in business

No	Area	Application exemplar	Value of application	Technologies	Source
1	E-commerce	Optimization of e-commerce platform	The structure of e-commerce websites can be optimized through AI and blockchain integration, whereby the application of blockchain technology solves the problem of cross border electronic payment, whereas the recommender system in e-commerce based on machine learning algorithms can help in online decision making.	• Blockchain • Deep learning • Neural network	Li et al. (2019)
2	Finance and accounting	Automated insurance system	An automated insurance system framework based on blockchain and extreme gradient boosting (XGBoost) machine learning algorithm can help to detect fraudulent claims, provide information about risky customers, and reduce monetary loss for the insurance industry.	• Blockchain • Machine learning	Dhieb et al. (2020)
		Credit evaluation system	A credit evaluation system based on blockchain and deep learning network can provide reliable information about transactions and credit evaluation of traders.	• Blockchain • Deep neural network	Mao et al. (2018)
		Financial portfolio optimization	Blockchain and neural network together helps in audit and secure settlement process, whereas deep reinforcement learning can enhance management and optimization of financial portfolio.	• Blockchain • Convolutional neural network • Deep reinforcement learning	Soleymani and Paquet (2020)
		FinBrain	Integration of technologies like AI, big data, blockchain technology, and cloud computing with finance can lead to automated and secure financial transactions.	• AI • Cloud computing • Blockchain • Big data	Zheng et al. (2019)
		Preventing corporate frauds	Integration of AI, blockchain, and smart contract can overcome the deficiencies of auditing and financial reporting and prevent corporate frauds caused by the failure of the auditor.	• Blockchain • IoT • Machine learning • Smart contract	Roszkowska (2020)
3	Healthcare	COVID-19 safe clinical practice	A generalizable predictive system that can contribute to controlling the pandemic risk and thus safeguarding both economic and public health.	• Blockchain • Machine learning	Fusco et al. (2020)
		GuardHealth	A data privacy preserving and sharing system that is based on a consortium of blockchain, smart contract, and a trust model implemented through graph neural network.	• Blockchain • Graph neural network • Smart contract	Wang et al. (2020)
		Hospital consolidation	A decentralized patient assignment system based on blockchain technology, machine learning, and integer programming that can enable healthcare providers to perform shared decision making by accessing the data about patients and collaborate with each other.	• Blockchain • Integer programming • Machine learning	Badré et al. (2020)
		Health data repository	A predictive model based on a machine learning classifier to help patients make data storage decisions in different types of blockchain-based data repository.	• Blockchain • Machine learning	Uddin et al. (2020)
4	Intellectual property right (IPR)	IPR management	AI and blockchain can be used to manage the IPR lifecycle, wherein blockchain-based solutions can be used for notarization of IPR assets, whereas machine learning-based data processing pipeline can be used to compare the IPR assets among competitors.	• Blockchain • Machine learning	Ragot et al. (2020)
		IPR management system	New IPR can be registered on a blockchain platform that can empower eligible stakeholders to use IPR data from the blockchain network, wherein text mining can help to identify the type of IPR for retrieval.	• Blockchain • Text mining, clustering, and classification	Alnafrah et al. (2019)
5	Management	Corporate online dispute resolution system	AI and blockchain integration can help parties of dispute to discover their own best/worst alternative to a negotiated agreement.	• AI • Blockchain	Barnett and Treleaven (2018)
		Corporate governance	AI can reduce reliance on humans for decision making in corporations, whereas blockchain can reduce the cost of voting and trade clearance by promoting direct shareholder requirement.	• AI • Blockchain • Distributed ledger	Bruner (2020)
6	Marketing	Customer satisfaction	A blockchain-based evaluation technique that can be used to provide a secure platform and that can predict customer satisfaction through the Long Short-Term Memory (LSTM) machine learning algorithm.	• Blockchain • Machine learning	Tian et al. (2020)
		Customer service	An open and automated customer service platform based on blockchain, IoT, and machine learning can enable small companies that do not have sufficient experience and data to automate their customer services without relying on third parties.	• Blockchain • IoT • Machine learning	Li et al. (2019)
7	Smart manufacturing	Cyber production system	A blockchain-enabled cyber production system can solve the problems of existing manufacturing practices when integrated with AI tools.	• AI • Blockchain	Lee et al. (2019)
		PriModChain	PriModChain integrates ethereum blockchains, federated machine learning, differential privacy, and smart contracts to improve the reliability and trustworthiness of IIoT data.	• Differential privacy • Ethereum blockchain • Federated machine learning • Smart contract	Arachchige et al. (2020)
		Production capability evaluation system	A production capability evaluation system based on blockchain, IoT, and machine learning can help to improve production efficiency.	• Blockchain • IoT • Machine learning	Li et al. (2019)
8	Social media	Controlling spread of false social media messages	Blockchain’s proof of work consensus algorithm can be used to reduce the spread of false information through social media, whereas parallel-dot-custom classifier of machine learning can be used to segregate the social media messages such as tweets as political and non-political.	• Blockchain • Machine learning.	Alagu Vignesh and Harini (2019)
		Secure instant messaging	A blockchain-based instant messaging scheme designed on Linux platform can be used to secure instant messaging, wherein machine learning algorithms detect anomaly in instant messaging by monitoring the activities on blockchain.	• Blockchain • Linux • Machine learning	Yi (2019)
9	Supply chain	Humanitarian supply chain	A framework integrating AI, blockchain, and 3D printing can improve the flow of products, information, and financial resources for humanitarian purposes.	• AI • Blockchain • Smart contract • 3D printing	Rodríguez-Espíndola et al. (2020)
		Smart farming	A platform based on the application of AI, blockchain, edge computing, and IoT can monitor the state of inventory in real time and ensure the traceability in the production process.	• AI • Blockchain • Edge computing • IoT	Alonso et al. (2020)
		Vaccine blockchain system	An intelligent system based on blockchain and machine learning can address the problem of vaccine record fraud and vaccine expiration in supply chains.	• Blockchain • Machine learning • Smart contract	Yong et al. (2020)
10	Transportation	Automation in airports	AI and blockchain enable airports to know their passengers’ preferences and to meet the need of the travelers in a better way.	• Blockchain • Predictive analytics	Mayer (2019)
		Railway asset management	Blockchain and big data analytics can be used for railways asset condition management.	• Descriptive analytics • Diagnostic analytics • Predictive analytics • Prescriptive analytics	McMahon et al. (2020)

The content analysis of the review corpus reveals that integrated AI and blockchain platforms have a wide range of applications for different areas in business, some of which are generic (e.g., management, marketing), and thus can applied across all industries, while some are specific to an industry (e.g., e-commerce, healthcare). In particular, the 10 areas unpacked through the analysis include e-commerce; finance and accounting; healthcare; intellectual property right; management; marketing; smart manufacturing; social media; supply chain; and transportation.

AI and blockchain are the key technologies propelling the wave of digital transformation. The convergence of both of these technologies can improve current business practices and introduce new business models that can act as independent economic agents making decisions autonomously. Blockchain can enhance transparency, trust, privacy, and security of business processes (Mao et al., 2018), whereas AI can detect patterns in data and optimize business practices (Salah et al., 2019). These two technologies are complementary by design and their true potential can only be unlocked if they are integrated (Sandner et al., 2020).

When AI and blockchain are used independently, they can cause series of concerns. On the one hand, AI suffers from issues related to trustworthiness, privacy, and explainability. On the other hand, blockchain experiences weaknesses such as security and scalability. The amalgamation of these two technologies can overcome these weaknesses and benefit businesses through secure data sharing and automatization of business processes (Sandner et al., 2020).

In recent years, numerous business cases of AI and blockchain integration have emerged. The confluence of AI and blockchain creates a highly trustworthy technology-enabled decision-making system that contributes toward a secure ecosystem for data exchange and transactions. Specifically, blockchain provides frictionless information access to AI models and helps to make accurate decisions in business.

The amalgamation of AI and blockchain technologies has also introduced decentralized autonomous business models that brings greater flexibility, agility, and cost-effectiveness to business. For example, Lee et al. (2019) introduce cyber physical system (CPS) for manufacturing industry, which facilitates self-optimizing, self-adjusting, and self-configuring production systems and solves the inadequacies of existing manufacturing processes. CPS has laid the foundation to build advanced production systems in which every functional element of the production chain such as design, manufacturing, supply chains, customer service, and support can be influenced (Lu, 2017).

AI and blockchain, with the hardware support of IoT, can also make supply chains more resilient and robust as products are traceable in real time throughout the supply chain (Alonso et al., 2020). Moreover, these technologies can improve the flow of products, information, and financial resources across supply chains (Rodríguez-Espíndola et al., 2020).

In accounting, blockchain can help prevent incorrect predictions generated by AI algorithms due to faulty data generation systems or tampering of data sources by authenticating the data generators. Specifically, AI, blockchain, and IoT can help companies to increase the quality of the audit process by improving the reliability of financial statements (Roszkowska, 2020), thereby overcoming audit-related problems and mitigating the risk of accounting fraud.

In finance, Irwin and Turner (2018) proposes an optimal solution for tracking illicit bitcoin transactions by using AI algorithms to analyze big data collected form bitcoin blockchain. Similarly, in insurance, extreme gradient boosting machine learning algorithm can detect fraudulent claims and risky customers by analyzing the data stored on blockchain (Dhieb et al., 2020). Noteworthily, blockchain can easily eliminate fraudulent practices in business and AI can create data classifiers and filters that makes it possible to verify the authenticity of the processes and users on a decentralized blockchain infrastructure (Salah et al., 2019).

In healthcare, blockchain protects privacy and increases security of health data (Wang et al., 2020). Integration of AI and blockchain create predictive system contributing to clinical workflow (Mamoshina et al., 2018). Using these technologies, patients can own and control their medical records.

In marketing, blockchain-based customer data acquisition can help companies to provide automated customer service. Li et al. (2019) propose an automated customer service platform based on machine learning, blockchain, and IoT, which helps small scale firms to provide high quality customer service without depending on third parties. This will eventually lead to higher satisfaction levels among the customers and improve the company’s profit. Moreover, customer satisfaction can be predicted using LSTM machine learning algorithm on a blockchain-based platform.

In social media, blockchain’s proof of work consensus algorithm can bring down the spread of false information (Alagu Vignesh & Harini, 2019) and machine learning algorithms can detect anomaly in instant messaging (Yi, 2019). Noteworthily, blockchain technology offers a potential solution to combat the increasing threat of misinformation in social media content (Christodoulou & Christodoulou, 2020). Specifically, blockchain holds the promise to restore trust in the digital ecosystem by offering greater transparency into the content lifecycle (Narayanan & Attili, 2021). Due to its decentralized nature, blockchain can track the digital journey of the content, verify its source, and check how it may have been manipulated. Additionally, a blockchain-based system backed by AI can authenticate the identity of the content creator and gauge his or her reputation for accuracy. This is supported by Christodoulou and Christodoulou (2020), who illustrated the implementation of a decentralized application built on Ethereum (blockchain) as a valuable tool for combating misinformation and fake news.

**Fig. 10:**
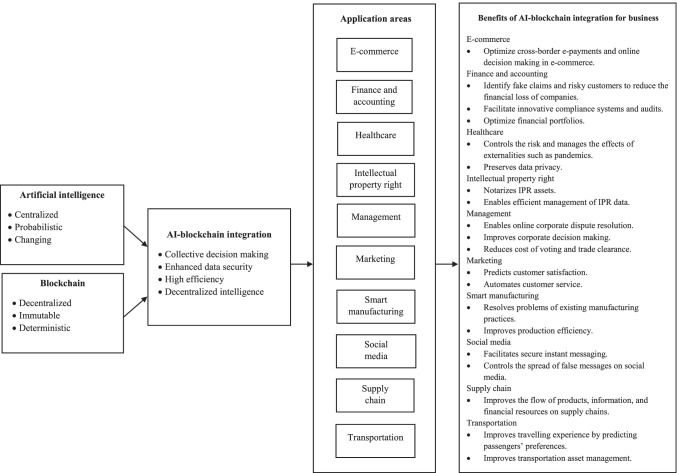
Framework for AI-blockchain integration in business

To this end, it is clear that the convergence of AI and blockchain can take place in multiple dimensions. Products, services, and business models can benefit from the integration of these technologies, wherein the convergence can digitally transform industrial corporations to drive the advancement of their business and pave their way into a new digital era (Makarius et al., 2020). Numerous exemplars exist for each area where AI and blockchain can be integrated for process improvement and value creation (e.g., asset management, customer service, dispute resolution, fraud prevention, production evaluation, supply chain monitoring). Moreover, the analysis shows that AI (e.g., edge computing, machine learning) and blockchain (e.g., distributed ledger, ethereum, smart contract) can manifest in numerous ways and that available proposals in the literature are highly technical and systems based (e.g., cyber production system, production capability evaluation system). More importantly, the review indicates that each application exemplar is supported with empirical and pragmatic evidence demonstrating its effectiveness and value, and as a whole, making a strong case on the valuable promise that AI and blockchain integration hold for business. Figure 10 presents the framework showing integration of AI and blockchain and highlights the application areas and benefits of integrated AI-blockchain platforms.

## Conclusion

AI and blockchain are two of the hallmark technologies of IR 4.0. Although the two technologies are relatively different in their own ways, the subject of amalgamation has received interest among scholars and professionals alike. Yet, there is a scarcity of research that consolidates and examines the state-of-the-art insights on the applications of integrated AI and blockchain platforms in business. The present study sought to address this gap through a bibliometric-content analysis, and in doing so, provides five key takeaways.

First, the study clarifies that AI and blockchain are IR 4.0 technologies that were introduced in 2016, with early research that integrates the two technologies for business emerging in 2017 but proliferated exponentially only from 2019 onwards. Here, the proliferation of research witnessed reaffirms the study’s contention that AI and blockchain integration holds immense promise for business applications.

Second, the study identifies Mamoshina et al.’s (2018) article on the convergence of AI and blockchain in healthcare, Liu et al.’s (2019b) article on performance optimization for blockchain-enabled IoT systems, and Mao et al.’s (2018) article on credit evaluation system based on LSTM and blockchain as the most influential articles on AI and blockchain integration for business. It is important that prospective authors are cognizant of the seminal articles in the field in order to position and design future research in novel ways to extend the knowledge of prior research.

Third, the study reveals that “*blockchain* and *machine learning*”, “*blockchain* and *smart contract*”, and “*blockchain* and *security*” are the most popular topics for research, and the revelation of “*blockchain* and *machine learning*” as the sturdiest association in the corpus is another source of triangulation to highlight the promise and importance of AI and blockchain integration in business, wherein machine learning is the AI technology that seems to be most relevant and sought after for business endeavors.

Fourth, the study unpacks four major thematic clusters that underpins the intellectual structure of research relating to AI and blockchain integration for business, namely *IR 4.0 and supply chains*, *smart healthcare*, *secure transactions*, and *finance and accounting*. Here, two clusters are generic and thus transcend across industries (i.e., *IR 4.0 and supply chains* and *secure transactions*) and two clusters are specific and thus industry focused (i.e., *smart healthcare* and *finance and accounting*). The small number of major thematic clusters is an indication that research on AI and blockchain integration for business is relatively new, and thus, research that spurs its continued growth to enrich existing clusters and to propel the emergence of new clusters is highly encouraged.

Fifth, the study reveals 10 areas for AI and blockchain integration in business suggested by existing studies in the field, namely e-commerce; finance and accounting; healthcare; intellectual property right; management; marketing; smart manufacturing; social media; supply chain; and transportation, wherein exemplars of integrating the two IR 4.0 hallmark technologies for business endeavors are offered alongside its potential value and sources.

To this end, this article has contributed in four major ways. First, this article has mapped the publication productivity of AI and blockchain integration for business, showing that the field remains in its infancy and has tremendous scope for growth and future research. Second, this article has identified the most influential articles in the field, thereby laying the foundation for future research to build on the seminal insights curated herein. Third, this article has unpacked the most prominent topics and themes on AI and blockchain integration for business, which enables prospective authors to gain a one-stop state-of-the-art insights of literature in the field. Fourth and finally, this article has revealed 10 application areas for AI and blockchain integration in business, which should be useful for business professionals who wish to leverage on scholarly research to identify areas for business transformation using the two IR 4.0 technologies, including exemplars that they could follow.

This article also delivers important implications to different stakeholders, including business managers, AI and blockchain developers, information technology (IT) vendors, and future scholars. For business managers, the knowledge about the potential of integrating AI and blockchain in business can encourage them to develop and implement projects utilizing both these technologies to foster resilient business operations and improve the performance of firms (Fosso Wamba & Queiroz, 2021; Rubin et al., 2021). AI and blockchain developers also stand to gain acute insights on how integrating these two technologies creates business synergy. Therefore, AI and blockchain developers can collaborate to develop automated and decentralized business applications that offer better governance, higher performance, and greater security of user confidentiality and privacy (Siala & Wang, 2022). IT vendors will also be able to better identify the market for AI and blockchain business solutions and position the value they bring through the identified areas for AI and blockchain integration in business. Finally, future scholars will be able to advance knowledge in the field by building on the current overview of AI and blockchain integration in business and venturing into new areas that would create new streams or enrich existing streams of research in the field. In this way, this article empowers both professionals and scholars with state-of-the-art insights so that they will be aware of the opportunities for AI and blockchain integration to enhance the growth, resilience, and robustness of business practices.

Nonetheless, this article, like any others, concedes its limitations. First, the data in this article is bounded by the accuracy and completeness of its source, and in this case, Scopus. It is important to note that Scopus, as a scientific database, was not developed for bibliometric analysis, and thus, may contain (unintended) errors. To mitigate potential (unintended) errors, the authors have ensured that they cleaned the bibliometric data retrieved from Scopus to the best of their ability, wherein duplicates and erroneous entries are removed, as recommended by Donthu et al. (2021a). Second, AI and blockchain integration is a dynamic field of research and a radical transformation in practice. In that sense, new innovations in its application and integration are expected, and thus, new streams of research are likely to proliferate rapidly. Therefore, prospective authors should not only rely on the review insights herein, but also to keep themselves updated with the latest research in the field, which can be done by using the search string herein this study.

Moving forward, prospective authors interested in conducting new research on the integration of AI and blockchain for business are encouraged to invest their efforts to uncover the application of such an integration from a business rather than engineering lens, as the study herein flagged the scarcity of insights emerging from business research. To fertilize such endeavors, this article calls for new research that answers the non-exhaustive research questions on AI and blockchain integration through a business lens in general and by cluster, which we summarize in Table 11.

**Table 11:** Future research questions on AI and blockchain integration through a business lens

Cluster	Future research question
All clusters	• What business activities and processes would benefit from AI and blockchain technologies, and to what extent would the integration of these technologies be of value to small, medium, and large enterprises?
	• What are the human characteristics and capabilities that nurture or prevent the effective implementation of integrated AI and blockchain innovations?
	• How can AI and blockchain integrated applications be diffused in ways that encourage adoption and that mitigate resistance?
	• What are the fundamental and value adding competencies and skills required to develop, implement, and manage AI and blockchain integrated systems, and how can human capital be reskilled or upskilled in order to meet these requirements?
	• How can the information or solutions generated from AI and blockchain integration impact into or inform managerial decisions, and what differences would explicit, implicit, intentional, and unintentional information or solutions produce?
	• What are the ethical issues and privacy rights that could transpire from AI and blockchain integration in business, and how can they be resolved?
	• What metrics can be used for business to monitor and manage the effectiveness of AI and blockchain integrated solutions?
	• How can business professionals and integrated technologies involving AI and blockchain co-exist and work together to create a better world?
Cluster 1: IR 4.0 and supply chains	• How can AI and blockchain integration be applied to curate and improve sustainable supply chains?
	• What is the impact of AI and blockchain integration on supply chain performance and relationships?
	• What is the impact of AI and blockchain integration in supply chains on economic, environmental, and social sustainability?
Cluster 2: Smart healthcare	• How can AI and blockchain integration be applied in omnichannel healthcare?
	• How can AI and blockchain integration be applied to manage economic and public health?
	• What is the impact of AI and blockchain integration in healthcare on economic and public health?
Cluster 3: Secure transactions	• How can AI and blockchain integrated systems be immunized against offline and cyber fraud?
	• What are the enablers and barriers to adopt AI and blockchain integration for secure transactions, how can the enablers be activated, and how can the barriers be mitigated?
Cluster 4: Finance and accounting	• How can AI and blockchain integrated systems be immunized against creative accounting?
	• What is the impact of AI and blockchain integration on revenue generation, and how can they be harmonized for cross-border financial transactions?

In short, this article makes clear that AI and blockchain integration is a global and legitimate phenomenon that holds tremendous promise for business, and that future research in this area should be enriched from the business perspective in order to complement the solutions offered through the engineering perspective and move the field forward. It is hoped that the insights herein this article will be useful for gaining a one-stop understanding of AI and blockchain integration as well as some prospective avenues for future research in the area through a business lens.
